# Use of social thermoregulation fluctuates with mast seeding and reproduction in a pulsed resource consumer

**DOI:** 10.1007/s00442-020-04627-7

**Published:** 2020-03-12

**Authors:** Thomas Ruf, Claudia Bieber

**Affiliations:** Department of Interdisciplinary Life Sciences, Research Institute of Wildlife Ecology, University of Veterinary Medicine Vienna, Savoyenstraße 1, 1160 Vienna, Austria

**Keywords:** *Glis glis*, Huddling, Kin selection, Local resource competition, Sociality

## Abstract

Edible dormice (*Glis glis*) can remain entirely solitary but frequently share sleeping sites with conspecifics in groups of up to 16 adults and yearlings. Here, we analysed grouping behaviour of 4564 marked individuals, captured in a 13-year study in nest boxes in a deciduous forest. We aimed to clarify (i) whether social thermoregulation is the primary cause for group formation and (ii) which factors affect group size and composition. Dormice temporarily formed both mixed and single-sex groups in response to acute cold ambient temperatures, especially those individuals with small body mass. Thus, thermoregulatory huddling appears to be the driving force for group formation in this species. Huddling was avoided—except for conditions of severe cold load—in years of full mast seeding, which is associated with reproduction and high foraging activity. Almost all females remained solitary during reproduction and lactation. Hence, entire populations of dormice switched between predominantly solitary lives in reproductive years to social behaviour in non-reproductive years. Non-social behaviour pointed to costs of huddling in terms of competition for local food resources even when food is generally abundant. The impact of competition was mitigated by a sex ratio that was biased towards males, which avoids sharing of food resources with related females that have extremely high energy demands during lactation. Importantly, dormice preferentially huddled in male-biased groups with litter mates from previous years. The fraction of related individuals increased with group size. Hence, group composition partly offsets the costs of shared food resources via indirect fitness benefits.

## Introduction

Animals may aggregate for various reasons, such as spatial resource distribution, for reasons of sexual selection and mating opportunities, or to lower individual predation risk, for instance by dilution effects (Hamilton 1971). Especially in small endotherms, another factor generating groups with tight contact is the reduction of thermoregulatory energy expenditure by huddling. In solitary rodents, huddles are often formed outside the reproductive season, when aggression is reduced (Gilbert et al. 2010).

In this regard, edible dormice (*Glis glis*) seem unusual, because these nocturnal, arboreal rodents, which have been reported to be both solitary and social (Čanády et al. 2016), form sleeping groups even when they are reproductively competent (Fietz et al. 2010). Given the aggression promoting effects of testosterone it is even more remarkable that, among reproductively active males, individuals with larger testes were found in larger groups than males with smaller testes (Fietz 2012; Fietz et al. 2010). From those previous studies (Fietz 2012; Fietz et al. 2010) it was not entirely clear, however, if social thermoregulation is the only, or even the primary force driving group formation in dormice. Individuals forming groups had lower body mass than solitary dormice (Fietz et al. 2010), which does point to huddling, as this behaviour reduces the increased heat loss of small endotherms. Low ambient temperatures in this study did not, however, lead to increased group formation, as would be expected from social thermoregulation. Also, up to now it is unclear whether adult dormice aggregate indiscriminately with unrelated conspecifics or prefer to nest with kin.

Further questions concerning group formation arise from the peculiar life history of dormice, which may entirely skip reproduction in years without high-caloric food resources. In reproductive years, dormice have a single litter born late in the summer, just in time with the ripening of beechnuts or acorn, which the young require for rapid growth and fattening in preparation for the 8-month hibernation season (Bieber 1998; Ruf et al. 2006). Hence, edible dormice are pulsed resource consumers (Ostfeld and Keesing 2000) that reproduce in full mast years, when all beech and oak trees produce seeds or in intermediate years, when at least a fraction of trees are seeding. In complete mast failure years, adults can sustain their lives and even gain weight, but forego reproduction (Bieber 1998; Pilastro et al. 2003; Ruf et al. 2006). This raises the question whether the mast seeding of trees and concomitant reproduction also affect group formation. These could be direct effects, if aggregations are involved in reproduction. Edible dormice are highly promiscuous with a high degree of multiple paternity of litters (Weber et al. 2018), which means that groups may offer opportunities for mating. Indeed, it has been suggested that enhanced prospects for mating and breeding are the main selective forces leading to animal aggregations in general (Wagner et al. 2000).

There also may be indirect effects via the impact of reproduction on energy expenditure. It is well known that in dormice, reproduction increases energy requirements in both sexes. Males as well as females lose weight early in the active season in reproductive, but not in non-reproductive years and females have high costs for lactation (Bieber 1998; Ruf et al. 2006; Zoufal 2005). This may, in turn, increase the necessity of energy savings, for instance, by increased huddling behaviour, which in dormice was estimated to amount to a reduction of metabolic rate by ~ 40% (Fietz et al. 2010).

To answer these questions, we analysed data collected over 13 years in a free-living population of edible dormice in the Vienna woods. We marked > 4000 individuals captured during fortnightly controls of nest boxes, which the animals use as sleeping sites in lieu of natural tree holes. We determined under which conditions adults and yearlings formed groups, excluding lactating mothers with their litters. We first analysed whether environmental factors, namely weather, tree seeding, and number of animals present, affect group formation in dormice. Second, we investigated which variables, both environmental and in terms of group composition, determine group size.

We hypothesized that if social thermoregulation was the primary reason for group formation, the frequency of group encounters should increase with decreasing environmental temperature, and possibly also increase with high precipitation. We second hypothesized that if group formation was instead primarily, or even solely caused by creating mating opportunities, it should be most pronounced in reproductive (full mast and intermediate) years. These two explanations—thermoregulatory and reproductive benefits—are not, however, entirely mutually exclusive. We finally hypothesized that, irrespective of the principal reason for aggregations, likely costs of group formation—such as sharing of mating opportunities or of food resources—may be lessened by preferably nesting with kin, due to indirect fitness benefits.

## Methods

### Study site and measurements

The study site was located close to St. Corona in the Vienna Woods (Lower Austria, 48°05′N/15°54′E; 400–600 m asl). The area (size ~ 15 km^2^) is covered by a mixed forest with most of the site dominated by deciduous beech (*Fagus sylvatica*, ~ 60% of the trees) and ~ 30% coniferous trees.

We checked a total of 211 nest boxes (volume ~ 6.5 l; mounted on trees at 2–4 m height) along forest trails at a mean distance of ~ 115 m, as determined by GPS (Cornils et al. 2017). The boxes were approximately evenly distributed over the study site. Nest boxes were inspected for the presence of edible dormice every 2nd week (mid-April to mid-November, 2006–2018). In 2011, the number of nest boxes checked was reduced to 124, disregarding boxes with very low capture rates. During the active season (April–November), edible dormice use these nest boxes (in place of natural tree holes in primeval forests) to rest during the day and raise their young. Every newly captured dormouse was marked with a subcutaneously injected PIT tag transponder (BackHome BioTec^®^, 13.8 mm × 2.1 mm or Tierchip Dasmann^®^, 8 mm × 1.4 mm). All dormice were sexed and classified as either juvenile (before the first hibernation), yearling (before the second hibernation period) or adult (after the second hibernation period) using fur colour and size as described in Schlund (1997). During each capture, dormice were weighed to the nearest gram. It should also be noted that there is no sexual size dimorphism in edible dormice (adult males 98.5 ± 0.9 g, adult females 97.6 ± 1.4 g; *n* = 923; means ± SEM over the active season in non-reproductive years; see also (Čanády et al. 2016).

Reproductive state in males was assessed from determining whether the testes remained in the regressed state (like during hibernation) and hence were not palpable. Even in reproductive years, the fraction of males with palpable testes never reached 100%, because the classification included times very early or late in the active season when gonadal involution was still present, or already under way. Dormice spend winters solitarily in underground hibernacula (Vietinghoff-Riesch 1960).

All captured dormice were returned to their nest boxes immediately after the measurements. To avoid major disturbances of mothers with small young (< 15 days), which could have led to infanticide or abandoning (Koenig 1960), we only recorded the mother’s ID and litter size in these cases. Juveniles were marked with transponders shortly after they opened their eyes, at ~ 3–4 weeks of age. These procedures were discussed and approved by the institutional ethics and animal welfare committee in accordance with GSP guidelines and national legislation in Austria.

The degree of beech mast was determined by the amount of pollen in the air close to the study site (gravity traps; Litschauer 2001) as well as by visual observation of beechnut abundance at the study site. Years of complete mast failure were also years of reproduction skipping. There were no juveniles encountered in 2010, 2012, 2014 and 2017. In all other years, we marked 78–639 juveniles per year.

### Weather data

Ambient temperature (*T*_a_) was recorded at hourly intervals at ~ 2 m height in the shade in the centre of the study site, using temperature loggers (iButton, DS1922L, Maxim, Dallas, USA; accuracy: ± 0.5 °C). Precipitation was estimated from data of a weather station located 30 km to the east of the study site (Hohe Warte), obtained from https://rp5.ru/Wetterarchiv_in_Wien,_Hohe_Warte_(Wetterstation), retrieved on 11-Jan-2019. For each capture day, we computed mean and minimum daily *T*_a_ and mean precipitation for the interval 12:00 h on the day prior to capture to 12:00 h on the day of capture. This interval was chosen to cover the entire night prior to nest box controls, when nocturnal dormice are active, and in early morning choose a nest box to spend the day in. Further, we also computed mean *T*_a_ for the hours 03:00–06:00 on early morning of the capture day, i.e. approximately the time when dormice decide which nest box to enter. We also computed daily mean and minimum *T*_a_ as well as the mean amount of precipitation for the last 3 as well as 7 days prior to nest box controls, to evaluate possible longer-term weather effects. Finally, we computed deviations of the above *T*_a_ variables from smooth splines through their long-term (13 year) means. This was done to see if these deviations (e.g. unseasonably cold days) would explain the formation of groups better than absolute *T*_a._

### Nest box occupancy

From a list of captures of individuals (*n* = 10,752 over 13 years), we first assembled a table of littermates and mothers of juveniles, i.e., lactating females in nest boxes with newly marked young. Out of 300 cases of nest boxes with juveniles, 271 were encountered with one adult or yearling female. In 29 cases, two lactating females were found in a nest box together with a litter. Co-nesting females were previously shown to be close kin (prevalently mother–daughter pairs; Marin and Pilastro 1994). Mean litter size was 5.9 when one female was present, and 9.2 when two females were present. Thus, it appears that in most of the cases of two females in a nest box (10% of cases), there were two litters. The juveniles marked at a body mass of ~ 20–30 g shortly after eye opening (i.e., day 21) and before the age of weaning (i.e., week 6, Koenig 1960), and the adult female from the same nest box were classified as related dormice. Given their age, we could be certain that all of these juveniles were offspring of the single lactating female present in the box, which was apparent in most cases from visibly enlarged nipples. In the 10% of cases when two females were present, it is most likely that all animals were also closely related (because communally breeding females are close kin; Marin and Pilastro 1994), despite the fact that there are high rates of multiple paternity in this population (Weber et al. 2018). Typically, juveniles that we considered kin were marked during their second encounter in biweekly checks. All juveniles marked at a later age, after weaning, were not included in the present analysis.

Single females with young were still considered “solitary” for the purposes of this study, because they did not form groups with other adult or yearling dormice. Almost always (97%) there were no males present together with juveniles. Only on 10 occasions, 1–2 adult or yearling males were found in the same nest box, but this was only the case when juveniles had reached an age well past weaning. Therefore, these males were not considered related. Second, we assembled a list of known other relatives of each juvenile, i.e., offspring of the same putative mother in a prior or later year. Subsequently we removed all captures of juveniles from the data set, because their mothers, rather than themselves, had chosen the nest box they were born into. Hence, unless stated otherwise all means, proportions and statistical results refer to yearling and adult dormice only.

We computed the number of dormice encountered per nest box and capture day, as well as the proportion of kin present (littermates, mothers, and potential aunts/grandmothers), the proportion of yearlings, the sex ratio, the proportion of males with palpable testes (among all males in a nest box), and the mean body mass of dormice in a nest box. To adjust for a likely effect and seasonal change of the total number of yearlings/adults present at the study site on the formation of groups, we also computed the number of dormice encountered in nest boxes for each 2-week capture period. All calculations were carried out using R 3.6.0 (R Core Team 2019).

### Statistical analyses

Our observation units were nest boxes, which are known to highly vary in their occupancy by dormice. This is because nest box locations differ in their attractiveness for dormice, mainly due to composition and other characteristics of the surrounding forest (Cornils et al. 2017). Therefore, we adjusted for variance caused by these factors by including nest box ID as a random intercept in mixed effects models, computed using function glmer from R-package lme4 (Bates et al. 2015). We used binomial mixed models to see if any external variables (i.e., *T*_a_, mast, dormouse numbers) would predict the formation of groups (i.e., more than one animal per nest box). Subsequently, we used mixed effects models for Poisson-distributed data to identify both environmental variables and group composition variables (i.e., sex ratio, proportion of related dormice, mean body mass) that had an effect on group size. Because visual inspection indicated that proportions of males as well as mean body mass changed non-linearly with group size, these variables were converted to two-level factors, coding for above and below median values. The proportions of yearlings and of males with palpable testes were not included as predictors in these models, because they could not be clearly differentiated from mast seeding (and reproductive) years: there are no yearlings in full mast years, because they always follow mast failure/non-reproduction years, and the proportion of males with palpable testes is always higher in intermediate and full mast years.

In both model types (binomial and Poisson), we always included the total number of adult dormice known to be present on the study site as a covariate. Starting with the full model (all main variables and all two-way interactions), we determined the best model by excluding predictors that did not improve AIC. Because of high multicollinearity, late night, daily, 3-day, and 7-day means of *T*_a_ and precipitation, as well as deviations from long-term means, were not entered simultaneously, but alternatively into the models. We found no evidence for overdispersion of data [dispersion parameter always ≤ 1.05; package blmeco (Korner-Nievergelt et al. 2015)]. Following Zuur et al. (2009) we subsequently used function Anova from package car (Fox and Weisberg 2011) to see if predictors significantly contributed to the variance explained by the best model.

To test if the number of related animals (littermates and mothers) encountered in the same nest box as yearlings or adults was greater than expected by chance we used a randomization procedure. After determining the number of related animals in each actual group, we took random samples of individuals occupying nest boxes in the same area and in the same year to create pseudo-groups of the same size. To adjust for an increased likelihood of related individuals staying in the same area (and being overrepresented in huddling groups by chance), random group formation was focused on the areas surrounding of the actual groups. Starting with neighbouring nest boxes, animals for random groups were sampled from boxes with increasing distance along forest trails (in alternating directions) until the sample size of the actual group was reached. The mean largest distance from which animals for random groups were sampled was 1.8 nest boxes, i.e. ~ 200 m to either side of the focal group. The proportion of related individuals among those random groups was taken as the null hypothesis and tested against the actual proportion of related animals.

## Results

### Group formation

During 4213 nest box controls, more than one dormouse was encountered in the same nest box in 1346 cases (32%). These groups were partly composed of mixed sexes (45%), but 36% were pure male, and 19% pure female groups. The number of males per group ranged from 0 to 9, as did the number of females per group. In 305 cases, females were found in groups together with males and had a litter later in the same active season, which renders groups mating opportunities.

Dormouse groups were short-term aggregations: the identical group of individuals was recaptured only on 158 occasions during fortnightly checks (11.7%), typically in the same or a neighbouring nest box in the same year, occasionally in 2 subsequent years. One couple of females was recaptured ten times over 2 years, which represented the most stable group. The largest group of dormice encountered twice consisted of four females, no larger group was ever found more than once.

**Fig. 1:**
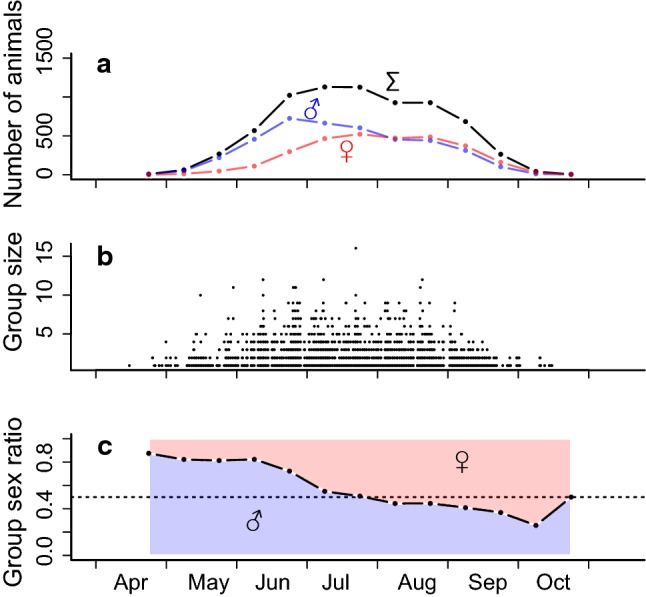
Seasonal pattern of **a** numbers of yearling or adult dormice present at the study site **b** group size (size 1 are solitary animals) and **c** sex ratio within groups at biweekly intervals. Pooled data from 13 study years. The blue and red lines in (**a**) show numbers of captured males and females, respectively. The blue and red areas in (**c**) show proportions of males and females, respectively, in dormouse groups (colour figure online)

Group formation was partly driven by the number of dormice active above ground (Table 1, Fig. 1). The frequency and size of groups changed seasonally and peaked in midsummer, along with the number of dormice captured on the site (Fig. 1a, b). Because male dormice on average emerge earlier from hibernation, the sex ratio (see also below) was particularly male biased early during the active season both among all captured individuals (Fig. 1a) and within nesting groups (Fig. 1c).

**Table 1:** Analysis of deviance table for the best mixed binomial model on the formation of groups of dormice

Term	$\chi^{2}$	*df*	*P*
Number present	156.1	1	< 0.001
Temperature	55.1	1	< 0.001
Mast	137.8	2	< 0.001
Number: mast	23.7	2	< 0.001
Temperature: mast	18.8	2	< 0.001

The model contained nest box ID as a random factor and correctly predicted 75% of actual groups versus solitary animals

The analysis of deviance was calculated using type II Wald $\chi^{2}$ tests. For each fixed effect tested the $\chi^{2}$ value, degrees of freedom (*df*) and *P* value are given

**Fig. 2:**
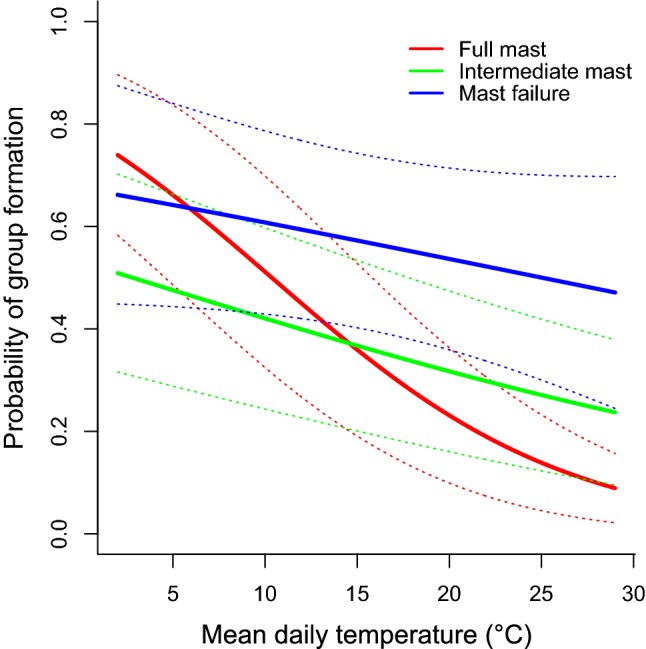
Binomial mixed model predictions (solid lines) and 95% confidence intervals (dotted lines) for the effect of mean daily ambient temperature on the probability of group formation in dormice. The effect of temperature was more pronounced in full mast years of beech trees compared with intermediate, and mast failure years

The formation of groups was also strongly affected by environmental temperature and beech mast (Table 1, Fig. 2). The probability of group formation increased with decreasing mean daily *T*_a_. Replacing mean daily T_a_ and precipitation data by 3-day or 7-day means, or by late night (03:00–06:00 h) means or minimum *T*_a_ on the previous day, did not improve the model (all AIC increased). Also, replacing *T*_a_s by their deviation from long-term means, to test if animals responded to unseasonably cold temperatures rather than absolute *T*_a_, also did not improve the best model.

The effect of *T*_a_ was most pronounced in full mast years, whereas the tendency for group formation was generally higher in mast failure years (Fig. 2). Also, the positive effect of dormouse numbers on group formation was more pronounced in full mast years (Table 1). Overall, the probabilities of encountering groups were 0.19, 0.32, and 0.50 in full mast, intermediate, and mast failure years, respectively. Expectedly, the proportion of males with palpable testes at any time during the active season (both solitary and in groups) was lowest in mast failure years (0.29 ± 0.016) and higher in intermediate (0.71 ± 0.011) and full mast years (0.64 ± 0.019). Among dormice encountered in groups, these proportions were almost identical (0.28 ± 0.019, 0.73 ± 0.016, 0.63 ± 0.032 for full, intermediate, and failure years, respectively).

There was a large variation between nest boxes in the frequency of their use. While some nest boxes were never used or only once, certain boxes were occupied up to 40 times by single animals over the study period, and up to 62 times by dormouse groups. A high positive correlation (Spearman’s $\rho$ = 0.75, P < 0.001) indicated, however, that single occupants and dormouse groups had the same preferences for, and dislikes of, certain nest boxes.

### Group size

**Fig. 3:**
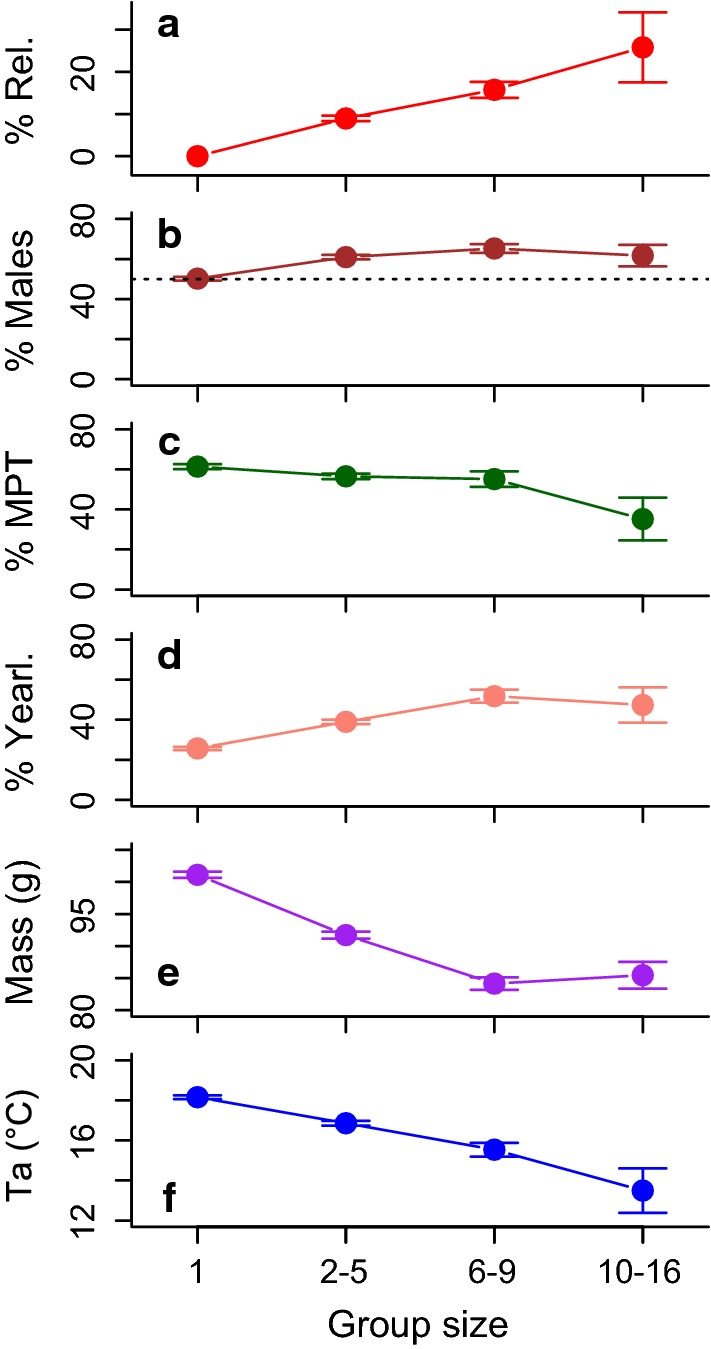
The association between group size (1 = solitary dormice) and group composition (**a**–**d**), as well as mean body mass (**e**) and mean daily ambient temperature (**f**). **a** Percentage of related dormice in the group. **b** Proportion of males. **c** Proportion of males with palpable testes among all males. **d** Proportion of yearlings in the group. Means ± SEM

**Fig. 4:**
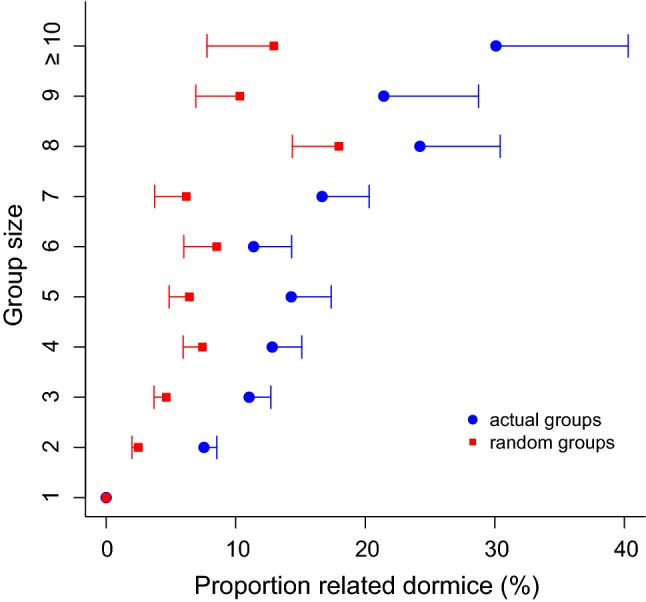
Group size as a function of the proportion (means ± SEM) of related dormice present (littermates and/or mothers from previous years). Blue circles: actual groups; red squares: simulated random groups (same number and size as actual groups), comprised animals present in nearby boxes in the same year. These random animals from neighbouring boxes had higher proportions of kin (5.7%) than random pseudo-groups generated by sampling dormice over the entire study site (0.9%). However, the proportion of related animals in actual groups was significantly higher (12.2%) than expected from random group formation, even when only the neighbourhood was considered ($\chi^{2}$ = 107.5; *P* < 0.001) (colour figure online)

Group size ranged from 2 to 16 individuals and decreased exponentially from 704 cases of 2 dormice down to 12 cases of 10–16 dormice. Group size was associated with group composition, mean body mass of group members, and *T*_a_ (Table 2, Fig. 3). Group size significantly increased with the proportion of related individuals present (Fig. 3a). This association was not coincidental, because the proportion of relatives was about twice as high than expected from random group composition (Fig. 4). In the majority of cases (61%) these relatives were all siblings, without their mother. The number of half-siblings (offspring of the same mother from other years) in groups was negligible. Either one or two putative half-sibs were found in groups only in 17 cases of nest box controls (0.04%).

**Table 2:** Analysis of deviance table for the best mixed Poisson distribution model on sizes of dormouse groups

Term	$\chi^{2}$	*df*	*P*
Number present	110.2	1	< 0.001
Proportion related	150.9	1	< 0.001
Proportion males	1.5	1	0.21
Body mass	102.5	1	< 0.001
Temperature	40.3	1	< 0.001
Mast	69.4	2	< 0.001
Number: temperature	6.9	1	< 0.01
Number: body mass	5.9	1	0.014
Body mass: proportion of males	10.7	1	0.001
Proportion related: proportion of males	8.2	1	< 0.01

The model contained nest box ID as a random factor and correctly predicted 62% of actual group sizes (range 1–16)

Increasing group size was also associated with slightly increased proportions of males, especially males with tangible testes (except for very large groups), yearlings, and low mean body mass (Fig. 3). In addition, mean group size was highest in mast failure years (3.5 ± 0.1), lower in intermediate years (3.0 ± 0.06) and lowest in full mast years (2.7 ± 0.1).

This positive effect of mast failure on group size was confirmed by the best mixed model, in which mast was the only main factor not involved in interactions (Table 2). The mean number of animals per nest box (including solitary animals) was 1.52 ± 0.02 (range 1–12) in mast seeding/reproductive years and 2.25 ± 0.06 (range 1–16) in mast failure, non-reproductive years. Mixed modelling revealed a number present: temperature interaction due to a stronger positive effect of high numbers when *T*_a_ was low. A high number of dormice present also had a stronger effect on group size when mean body mass was low. The third interaction was caused by the fact that body mass had a stronger impact when the proportion of males was low, suggesting a more pronounced response of females to low body mass. Finally, a high proportion of related animals present had a greater effect on group size when the fraction of males was large.

Group size increased with decreasing *T*_a_, depending on the number of dormice on the site (Fig. 3, Table 2). Again, using 3-day or 7-day means of weather data or minimum instead of mean daily *T*_a_, did not improve the model. Also, the model was not improved by including precipitation.

### Sex ratios

At the time of weaning, there were more male than female juveniles (sex ratio 1.4; $\chi^{2}$ = 61.0; *P* < 0.001) on the study site. However, in reproductive years the sex ratio among yearling and adult dormice encountered solitarily was 0.91, slightly biased towards females ($\chi^{2}$ = 4.6, *P* = 0.03), but heavily male-biased in groups (ratio 1.7; $\chi^{2}$ = 178.1; *P* < 0.001). In non-reproductive years, sex ratios were equally male biased among solitary animals (ratio 1.8; $\chi^{2}$ = 33.4; *P* > 0.001) and dormouse groups (ratio 1.7 $\chi^{2}$ = 92.5; *P* < 0.001).

## Discussion

The average *T*_a_ during the previous 24 h was a decisive factor in driving the formation of dormouse groups and the frequency of group formation increased in the cold under all conditions of tree seeding (Fig. 2, Table 1). Further, group size was higher at low *T*_a_, especially when many dormice were present (Fig. 3, Table 2). Also, dormice forming groups had smaller body masses and thus lower energy reserves and higher rates of heat loss (Fietz et al. 2010; Schaefer et al. 1976). Hence, there can be little doubt that huddling in response to cold exposure was the primary reason for aggregations. We cannot rule out that groups also provided mating opportunities. However, our finding that both group formation frequency (Fig. 2) and group size was highest in non-reproductive/mast failure years argues against a prominent role of reproduction in group formation. Interestingly, in reproductive/full mast years, dormice even appeared to avoid group formation until forced to by low *T*_a_ (Fig. 2). This pattern was not only due to females turning solitary when they had litters, because it also occurred when females were disregarded. Thus, avoidance of groups in full mast years may reflect increased competition for food resources in the areas surrounding nest boxes. This competition has been, of course, identified as a potential cost of aggregations in various species (e.g., Clutton-Brock and Huchard 2013).

The fact that group formation and group size was enhanced by high dormouse numbers may suggest that nest boxes are a limited resource in this population living in a working forest, in which natural tree holes are probably scarce. Still, 4% of nest boxes were never used by dormice at all and 24% were never inhabited by groups, indicating that the attractiveness of nest sites strongly varied. A previous study on the same population has revealed that it is mainly a high diversity of feed plants as well as predator-avoiding features, such as a high degree of canopy closure and vertical stratification of the forest, which makes areas surrounding certain nest boxes more attractive than others (Cornils et al. 2017). As indicated by a high correlation ($\rho$ = 0.75) between occupancy rates there was no principal difference, however, in preferences for certain nest box locations between single occupants and groups.

Effects of the number of dormice present, and their sex specificity, may also explain the male-biased sex ratio within groups, at least partly. There was a seasonal change in the sex ratio at the site, due to males emerging earlier from hibernation (e.g., Bieber 1998), which largely explains a similar change within groups (Fig. 1a, c). Also, the sex ratio at the study site was generally male biased (ratio 1.4) at weaning. A similar male bias was found in other dormouse populations (Koppmann-Rumpf et al. 2015; Schlund et al. 2002), but not at all locations (Burgess et al. 2003; Kryštufek et al. 2003). In a German population, there was evidence for increased mortality among juvenile males, which led to the hypothesis that this is compensated by a male-biased offspring ratio (Koppmann-Rumpf et al. 2015). There was no sign, however, of higher mortality among juvenile males at our study site nor were there noteworthy sex-specific differences in adult survival rates in several populations across Europe (Lebl et al. 2011). Notably, there was no sexual size dimorphism in adults (see Methods), and no sign of effects of female condition, i.e., body mass, on offspring sex ratio (c.f., Trivers and Willard 1973). Therefore, we hypothesize that the male-biased sex ratio at weaning (and most likely at birth) in our population was due to local resource competition (Clark 1978; Silk and Brown 2008). In dormice, females are philopatric and—apparently unlike males—defend territories during reproduction (Cornils et al. 2017; Ściński and Borowski 2008; Vietinghoff-Riesch 1960; Weber et al. 2018). As in most mammals, juvenile dormouse males are the class most prone to disperse over larger distances to areas outside the mothers’ territory (Cornils et al. 2017). These are all conditions which should select for females reducing local competition with female kin by adjusting their offspring sex ratio towards non-territorial males. However, at our study site at least a fraction of the juvenile males stayed in the area and were recaptured in groups with litter mates in subsequent years. This behaviour contributed to a significant surplus of males in dormice groups. Thus, resource competition was not completely avoided, but diminished, because the most intense competition will occur between females, which have extremely high-energy demands during lactation (Hammond and Diamond 1997; Zoufal 2005). Also, the costs of sharing food trees with group members were apparently offset by energy savings via huddling. Further, resource competition in large huddles arguably was limited by the fact that large groups mostly consisted of relatively young, small individuals (Fig. 3d, e) with correspondingly lower energy demands.

Reproducing and lactating females, which are known to defend so-called breeding ranges (Vietinghoff-Riesch 1960), strictly avoided sharing of territories by remaining solitary. This behaviour led to an even sex ratio among solitary animals during reproductive years, despite the surplus of males at the study site. This finding shows that females are well able to defend and monopolise sleeping sites, if necessary. However, solitary nesting also occurred among males, especially those with large body mass (Fig. 3b, e). Interestingly, this evasion of resource competition appeared to present even during mast seeding, which may seem like a superabundant food supply. However, mast seeding trees in close proximity to the nest, especially at locations with relatively low predation risk (Cornils et al. 2017), may still be extremely valuable resources. Non-reproducing and smaller individuals, on the other hand, did tolerate—and likely profited from—the presence of conspecifics especially when they were small and exposed to cold.

In these cases, the benefits of huddling, namely local heating and reduced heat loss were often shared with previous litter mates, i.e., kin. Also, the costs of aggregations in terms of competition were mitigated by the fact that resources were also commonly shared with kin, which should lead to indirect fitness benefits. Since competition for resources will strongly increase with the number of individuals present, it seems highly adaptive that larger groups of dormice contained not just a constant fraction, but increasing proportions of relatives (Fig. 3a). The fraction of kin in groups may have been even underestimated, because mothers, offspring and siblings could be identified only if dormice were captured as juveniles before weaning. Another benefit of huddling with kin can be an increased stability of family groups, compared with groups of unrelated animals (Schradin et al. 2006), but it would require much more frequent nest box controls than could be carried out here, to see if this is also a factor in groups of edible dormice. Huddling with close relatives is widespread and particularly recognizable in pups huddling in a litter, e.g., in mice and rats, in cases of communal nesting, or in species permanently living in family groups, such as alpine marmots (review in Gilbert et al. 2010). Short-term sleeping/huddling groups of adult animals are frequently formed in response to cold, but these are mostly huddles of non-related individuals (e.g., Howard 1949; Radespiel et al. 1998). However, there are several species of otherwise ‘asocial’ squirrels (Koprowski 1996; Williams et al. 2013), as well as Japanese macaques (Takahashi 1997), in which the formation of temporary huddling groups of adults, similar to dormice, involves the preferential grouping of kin.

The exact proximate mechanisms of the establishment of huddling groups presently remain unclear. They likely, however, involve the recognition of familiar individuals after at least one hibernation season. Apparently, it is familiarity with individuals that were raised in the same nest box, rather than actual kinship, which leads to the high proportion of kin during group formation. Dormice may be actually unable to detect kin as such, because half-sibs from other years, i.e. unfamiliar kin, were not overrepresented in groups. Interestingly, the apparent ability to recognize litter mates in later years is in line with the finding that, while hibernation may impair the retention of conditioned tasks and spatial memory, it does not affect social memory (Millesi et al. 2001).

Previous studies on grouping in *Glis glis* have entirely focused on males and interactions with testicular function (Fietz 2012; Fietz et al. 2010), which may be interpreted to mean that this behaviour is a male trait. In spite of a certain male bias in group composition (Fig. 3b) this is clearly not the case, as 38.7 ± 1.0% of group members were females and 19% of groups consisted solely of females. It has been suggested that huddling is used in particular by sexually active males, because the alternative thermoregulatory mechanism of energy savings during the active season, i.e., torpor during the daily resting phase, is prevented by high levels of circulating testosterone (Fietz et al. 2010). Accordingly, edible dormice use torpor during summer almost exclusively in non-reproductive mast failure years (Bieber et al. 2017). However, torpor and huddling are not mutually exclusive. In fact, using continuous records of body temperature in a non-reproductive year (Bieber et al. 2017), we found several cases of dormice exhibiting torpor while resting in groups of 2–5 adults. Torpor and huddling may even have synergistic effects if the timing of entrance into, and rewarming from torpor are synchronized between individuals (e.g., Ruf and Arnold 2000). Not surprisingly then, huddling in Japanese field mice facilitates the use of daily torpor and further enhances energy savings (Eto et al. 2014). In line with possible complementary effects, huddling in dormice was much more likely to occur in mast failure years (Fig. 2), just like torpor (Bieber et al. 2017). Future investigations of the interaction between huddling and torpor will require detailed body temperature records in entire groups of dormice.

At present, it seems clear, however, that dormice massively switch from high-energy turnover, continuously high body temperature and intense foraging in reproductive years to an energy-saving mode in non-reproductive years that makes extensive use of huddling (Fig. 2), as well as reduced foraging activity, short torpor, and even long-term estivation (Bieber et al. 2017; Bieber and Ruf 2009; Hoelzl et al. 2015). Hence, the population-wide synchronization of reproduction in this species is associated with profound adjustments of behaviour, including altered group formation and sociality. It could almost be said that dormice switch from a solitary life in mast seeding/reproductive years to being social mammals in non-reproductive years. We are not aware of any other mammal showing similar shifts, on a population level, in their degree of sociality induced by environmental fluctuations.

## Data Availability

Upon publication of this article, data will be made available from the University of Vienna PHAIDRA data repository.
